# On Chronic Rheumatism

**Published:** 1807-11

**Authors:** 


### *To the Editors of the Medical and Physical Journal.*

Gentlemen,

CONVINCED that no arguments will be superfluous, no time mispent, nor a page of your Journal misapplied, by attempting to corroborate a practice, which is justly entitled to our warmest support, I take the liberty to transmit you the two following cases of the successful treatment of Chronic Rheumatism, by a plan suggested (in a former number) by Mr. Davies of Piccadilly. The symptoms of this disease are so generally known, and the proximate cause so accurately described by him to be, "an atony of the extreme vessels," that I should consider any comment on that head, not only unnecessary, but superfluous. However, as I am no advocate for practice without theory, for effects without causes, for the application of remedies, without some idea of the "*Modus operandi*," I beg leave to mention, that, what particularly recommends this mode of treatment is, that practice is here built upon principle, and that principle founded in reason. But to prevent any unnecessary intrusion on your valuable pages, I will, without any further introduction, proceed to the subject.

Mrs. M. was suddenly affected with the usual symptoms of "Rheumatismus Chronicus;" the disease was so extremely violent, that an unusual degree of palpitation was excited, excited, and the subject in the most excruciating pain. A desire to alleviate as much as possible the distresses of his patient, induced the medical attendant to endeavour to remove this most unpleasant sensation (the palpitation) by venæsection; accordingly, she was freely bled to the amount of about ten or twelve ounces; this, however, afforded only a temporary relief, and the symptoms soon returned with their accustomed violence. As the indication was so clear, no hesitation was entertained on the part of the practitioner, what mode of treatment to adopt. He had immediate recourse to *that* already hinted. With this intention, draughts composed of the decoct. cinchonæ and a little warm stomachic tincture, were given every four hours, while the exhibition of large doses of opium was regularly continued; and it is with a degree of satisfaction I have the pleasure to add, the result exceeded our most sanguine expectations. Nor was the terebinthinate application less regularly had recourse to, by a constant use of which, (in conjunction with the internal medicines) the patient was enabled, in the course of a few days, to walk across the room, which, till now, she had not done during her illness. The rapid strides she was apparently making towards a hasty recovery, did not act, as an inducement to discontinue those medicines, which had hitherto proved so unprecedently successful. It should have been remarked that, during the violence of the disease, the opium was *gradually* increased, as the constitution would admit, the limit of which amounted to 6 grs. in four-and-twenty hours, which quantity, I believe, it never exceeded. Every day now exhibited a more desirable appearance, so that nothing prevented me from pronouncing my patient *a complete convalescent*; nor would even this epithet be applicable to her long, for in the course of two or three weeks, I had the pleasure to find her (by a constant and regular perseverance in the plan prescribed) perfectly recovered; since which she has not been attacked with the least return of her disorder.

The second case was Mr. D. G. In order to avoid repetition, which must otherwise be the case, I shall only just observe, that the symptoms, in this instance, were just the same as in the former, except from the less violence of the attack, no palpitation was occasioned; on which account the necessity of venæsection was entirely superseded. In all other respects, the same mode of treatment *precisely* was adopted, and to my extreme satisfaction, with equal success. From the above two cases, with a few others, which which have lately fallen under my observation, I am so fully satisfied of the propriety of this treatment, that whenever a case of chronic rheumatism engages my attention, I cannot by any means be induced to adopt, or even think of, any other. It is not to be expected, that a practice, which has only been ushered into the notice of the medical public, by individual recommendation, will at once be received with general approbation; I therefore consider it the duty of every practitioner, to contribute all the support he is able, by giving every additional account of its successful result, which falls under the eye of his immediate notice, for I am fully convinced the wider the scale of its adoption is extended, the more the benefit of its results will be experienced.

I am, &c.

*Octeber* 9, 1807. AMICUS.

